# Tetraarsenic Hexoxide Induces Beclin-1-Induced Autophagic Cell Death as well as Caspase-Dependent Apoptosis in U937 Human Leukemic Cells

**DOI:** 10.1155/2012/201414

**Published:** 2011-09-08

**Authors:** Min Ho Han, Won Sup Lee, Jing Nan Lu, Jeong Won Yun, GonSup Kim, Jin Myung Jung, Gi-Young Kim, Su-Jae Lee, Wun-Jae Kim, Yung Hyun Choi

**Affiliations:** ^1^Department of Biochemistry, Dongeui University College of Oriental Medicine and Department of Biomaterial Control (BK21 Program), Dongeui University Graduate School, 42 San, Yangjung-don, Busan 614-052, Republic of Korea; ^2^Department of Internal Medicine, Institute of Health Sciences and Gyeongnam Regional Cancer Center, Gyeongsang National University School of Medicine, 90 Chilam-dong, Jinju 660-702, Republic of Korea; ^3^Department of Internal Medicine, Institute of Health Sciences, Gyeongsang National University School of Medicine, Jinju 660-702, Republic of Korea; ^4^Institute of Life Science and School of Veterinary Medicine, Gyeongsang National University, Jinju 660-701, Republic of Korea; ^5^Department of Neurosurgery, Institute of Health Sciences, Gyeongsang National University School of Medicine, Jinju 660-702, Republic of Korea; ^6^Laboratory of Immunobiology, Department of Marine Life Sciences, Jeju National University, Jeju 690-756, Republic of Korea; ^7^Department of Chemistry, Hanyang University, Seoul 133-791, Republic of Korea; ^8^Department of Urology, Chungbuk National University College of Medicine, Cheongju, Chungbuk 361-763, Republic of Korea

## Abstract

Tetraarsenic hexaoxide (As_4_O_6_) has been used in Korean folk remedy for the treatment of cancer since the late 1980s, and arsenic trioxide (As_2_O_3_) is currently used as a chemotherapeutic agent. However, evidence suggests that As_4_O_6_-induced cell death pathway was different from that of As_2_O_3_. Besides, the anticancer effects and mechanisms of As_4_O_6_ are not fully understood. Therefore, we investigated the anticancer activities of As_4_O_6_ on apoptosis and autophagy in U937 human leukemic cells. The growth of U937 cells was inhibited by As_4_O_6_ treatment in a dose- and a time-dependent manner, and IC_50_ for As_4_O_6_ was less than 2 *μ*M. As_4_O_6_ induced caspase-dependent apoptosis and Beclin-1-induced autophagy, both of which were significantly attenuated by Bcl-2 augmentation and N-acetylcysteine (NAC) treatment. This study suggests that As_4_O_6_ should induce Beclin-1-induced autophagic cell death as well as caspase-dependent apoptosis and that it might be a promising agent for the treatment of leukemia.

## 1. Introduction

Arsenic trioxide (As_2_O_3_), a component of Chinese medicine, has been successfully employed for the treatment of acute promyelocytic leukemia (APL) [1, 2] and it has recently been shown to have some efficacy against a certain type of solid cancers [3, 4]. It is taken parenterally via an IV drip. With regard to anticancer effects of As_2_O_3,_ many studies have shown that As_2_O_3_ is capable of inducing programmed cell death. There are two types of programmed cell death reported. One is apoptosis, type I programmed cell death which is characterized by a highly stereotypical series of morphological and biological changes, such as cytoplasmic shrinkage, blebbing of the plasma membrane, chromatin condensation, and DNA degradation [5]. Another is autophagy, type II programmed cell death [6]. Autophagy is originally named as a process of protein recycling. It begins with sequestering cytoplasmic organelles in a membrane vacuole called autophagosome, which are double-membrane cytoplasmic vesicles to engulf various cellular constituents, and to fuse with lysosomes, where the sequestered cellular constituents are degraded and recycled. 

Tetraarsenic hexoxide (As_4_O_6_) has been used as a Korean folk remedy for the management of cancer since the late 1980s because its toxicities were minimal compared to conventional cytotoxic chemotherapy. However, the anticancer effects of As_4_O_6_ have not been investigated much although the anticancer effects of arsenic trioxide (As_2_O_3_) have been investigated in many leukemic cells [7–9]. A comparison study of the anticancer effects between As_2_O_3_ and As_4_O_6_ demonstrated that As_4_O_6_ was more effective in suppressing human cancer cells *in vitro* and *in vivo,* and that As_4_O_6_-induced cell death pathway was different from that of As_2_O_3_ [10]. Upregulation of p53 and v-erb-b2 erythroblastic leukemia viral oncogene homolog 2 (ERBB2) was noted in As_4_O_6_-induced cell, but not in As_4_O_6_-induced cell death. In addition, As_4_O_6_ has been used orally, whereas As_2_O_3_ has been used as a parenteral drug. Oral agents are more convenient to take than parenteral agents. Hence identifying the molecular mechanisms involved in its anticancer effects would allow us to contribute to developing a new oral agent. Here, we investigated the mechanisms of anticancer effects of As_4_O_6_ in U937 human leukemic cells.

## 2. Materials and Methods

### 2.1. Cells and Reagents

U937 human leukemic cells from the American type culture collection (Rockville, MD, USA) were cultured in RPMI 1640 medium (Invitrogen Corp, Carlsbad, CA, USA) supplemented with 10% (v/v) fetal bovine serum (FBS) (GIBCO BRL, Grand Island, NY, USA), 1 mM L-glutamine, 100 U/mL penicillin, and 100 **μ**g/mL streptomycin at 37°C in a humidified atmosphere of 95% air and 5% CO_2_. The Bcl-2 overexpressing U937 cells were a generous gift from Dr T.K. Kwon (Department of Immunology, Keimyung University School of Medicine, Taegu, Republic of Korea) and were maintained in a medium containing 0.7 **μ**g/mL geneticin (G418 sulfate). As_4_O_6_ was obtained from Chonjisan institute (Seoul, Republic of Korea). Antibodies against Bcl-2, Bax, Bad, Bcl-xL, XIAP, procaspase 3, procaspase 8, and procaspase 9 were purchased from Santa Cruz Biotechnology (Santa Cruz, CA, USA). Antibodies against poly (ADP-ribose) polymerase (PARP), PLC*γ*-1, LC3, and Beclin-1 were purchased from PharMingen (San Diego, CA, U.S.A.). Antibody against *β*-actin was from Sigma (Beverly, MA). Peroxidase-labeled donkey anti-rabbit and sheep anti-mouse immunoglobulin and an enhanced chemiluminescence (ECL) kit were purchased from Amersham (Arlington Heights, IL). Caspase activity assay kits were purchased from R&D systems (Minneapolis, MN, USA.). All other chemicals not specifically cited here were purchased from Sigma Chemical Co. (St. Louis, MO). All these solutions were stored at −20°C. Stock solutions of DAPI (100 **μ**g/mL) and propidium iodide (PI, 1 mg/mL) were prepared in phosphate-buffered saline (PBS).

### 2.2. Cell Viability Assays

For the cell viability assay, the cells were seeded onto 24-well plates at a concentration of 5 × 10^5^ cells/mL and then treated with the indicated concentration of As_4_O_6_ for 24 h. MTT (0.5 mg/mL) was subsequently added to each well. After 3 h of additional incubation, 100 **μ**L of a solution containing 10% SDS (pH 4.8) plus 0.01 N HCl was added to dissolve the crystals. The absorption values at 570 nm were determined with an ELISA plate reader.

### 2.3. Nuclear Staining

After treatment with the indicated concentration of As_4_O_6_, the cells were harvested, washed with phosphate-buffered saline (PBS), and fixed with 3.7% paraformaldehyde in PBS for 10 minutes at room temperature. Fixed cells were washed with PBS and stained with 2.5 **μ**g/mL 4,6-diamidino-2-phenylindole (DAPI) solution for 10 min at room temperature. The cells were washed two times with PBS and analyzed by a fluorescent microscope.

### 2.4. Flow Cytometry Assay

The cells were plated at a concentration of 2 × 10^5^ cells/well in six-well plates. Reduced (sub-G_1_) DNA content was measured by PI staining. The DNA content in each cell nucleus was determined with a FACSCalibur flow cytometer (Becton-Dickinson, San Jose, CA, U.S.A.). Two independent experiments were performed [11].

### 2.5. Western Blotting

The cells were harvested and lysed, and protein concentrations were quantified using the BioRad protein assay (BioRad Lab., Hercules, CA, U.S.A.). The proteins of the extracts were resolved by electrophoresis, electrotransferred to a polyvinylidene difluoride membrane (Millipore, Bedford, MA), and then the membrane was incubated with the primary antibodies followed by a conjugated secondary antibody to peroxidase. Blots were developed with an ECL detection system.

### 2.6. Caspase Activity Assay

Caspase activity was determined by a colorimetric assay according to the manufacturer's protocol in a kit for caspase activity. In brief, the cells were lysed in the supplied lysis buffer. The supernatants were collected and incubated with the supplied reaction buffer containing dithiothreitol and substrates at 37°C. The reaction was measured by determining the change in absorbance at 405 nm using the microplate reader [12].

### 2.7. Quantification of Acidic Vesicular Organelles (AVOs) with Acridine Orange Staining

In acridine orange-stained cells, the cytoplasm and nucleolus fluoresce bright green and dim red, whereas acidic compartments fluoresce bright red. Therefore, we stained the cells with acridine orange for 17 min. Green (510–530 nm) and red (650 nm) fluorescence emission from 1 × 10^4^ cells illuminated with blue (488 nm) excitation light was measured with a a FACSCalibur flow cytometer (Becton-Dickinson, San Jose, CA, U.S.A.). Three independent experiments were performed.

### 2.8. Statistics

Each experiment was performed in triplicate. The results were expressed as means ± SD. Significant differences were determined using the one-way analysis of variance (ANOVA) with post-test Neuman-Keuls in the cases at least three treatment groups and Student's *t*-test for two group comparison. Statistical significance was defined as *P* < 0.05.

## 3. Results

### 3.1. Responses of U937 Human Leukemic Cells to As_4_O_6_

**Figure 1:**
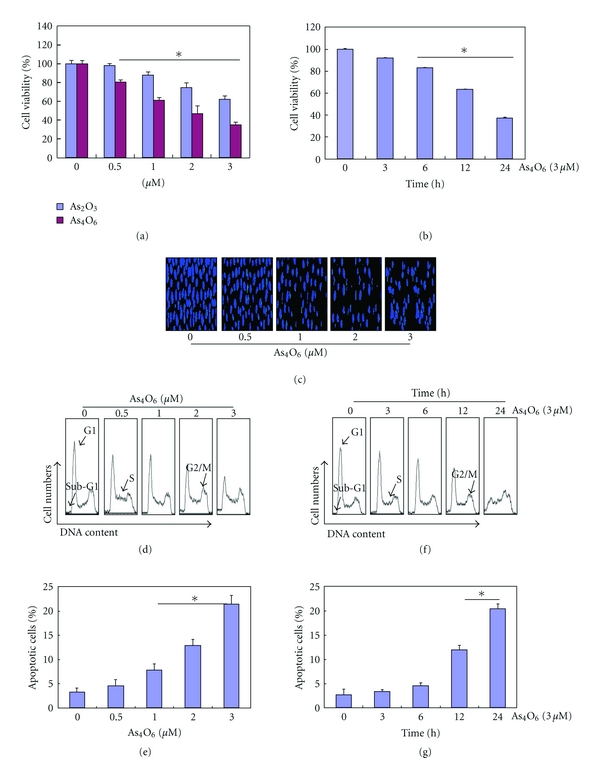
Inhibition of cell growth and induction of apoptosis by As_4_O_6_ in U937 cells. The growth inhibition and cytotoxicity As_4_O_6_ are a dose- and time-dependent manner. The efficacy of As_4_O_6_ is superior to that of As_2_O_3_. The cells were seeded at the density of 5 × 10^4^ cells per mL. The inhibition of cell growth was measured by MTT assay. (a) and (c) The cells were treated with the indicated concentrations of As_4_O_6_ and As_2_O_3_ for 24 hours. (b) and (f) The cells were treated with 3 **μ**M of As_4_O_6_ for the indicated times. The growth inhibition and cytotoxicity As_4_O_6_ are exhibited in a time-dependent manner. (c) After fixation, the cells were stained with DAPI solution to observe apoptotic bodies, which were more frequently seen in higher doses. Stained nuclei were then observed under fluorescent microscope using a blue filter (Magnification, X 400). (d)–(g) To quantify the extent of As_4_O_6_-induced apoptosis, sub-G1 DNA content, which represents the fractions undergoing apoptotic DNA degradation, was analyzed by flow cytometry. The data are shown as means ± SD of three independent experiments. *P < 0.05 between the treated and the untreated control groups.

To investigate the antitumor activity of As_4_O_6_, U937 cells were treated with various concentrations of As_4_O_6_ for 24 h. The cell growth was assessed by MTT assay. The MTT assay revealed that the growth of U937 cells was inhibited by As_4_O_6_ treatment in a dose- and time-dependent manner, and the 50% inhibition of cell growth (IC_50_) was less than 2 **μ**M (Figures 1(a) and 1(b)). The efficacy of As_4_O_6_ was superior to that of As_2_O_3_ in terms of growth inhibition (Figure 1(a)).

### 3.2. Effects of As_4_O_6_ on Apoptosis

To determine whether the decrease in viability of U937 cells was caused by the induction of apoptosis, we assessed the changes in nuclear morphology of As_4_O_6_-treated cells by DAPI staining. The DAPI staining revealed the condensed and fragmented nuclei at a concentration of 2 **μ**M or higher. This is usually witnessed in apoptosis (Figure 1(c)). To estimate the population of the cell death, we measured cells with sub-G1 DNA content by flow cytometry. A significant accumulation of cells with sub-G1 DNA content was noted in a dose-dependent (Figures 1(d) and 1(e)) and time-dependent manner (Figures 1(f) and 1(g)).

### 3.3. Caspases Activation and Subsequent Cleavage of Their Substrates by As_4_O_6_

**Figure 2:**
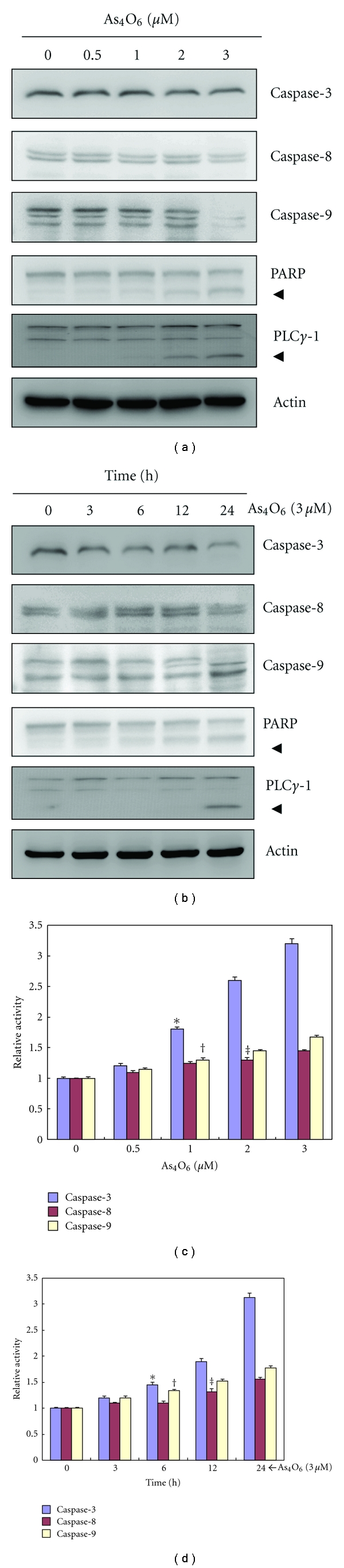
Activation of caspases and cleavage of PARP during the As_4_O_6_-induced apoptosis in U937 cells. The activation of caspases and cleavage of PARP by As_4_O_6_ are a dose- and time-dependent. (a) and (c) The cells were incubated at the indicated concentrations of As_4_O_6_ for 24 h. (b) and (d) The cells were treated with 3 **μ**M of As_4_O_6_ for the indicated times. (a) and (b) Total cell lysates were resolved by SDS-polyacrylamide gels and transferred onto nitrocellulose membranes. The membranes were probed with the anticaspase-3, anticaspase-8, anticaspase-9, and anti-PARP antibodies. The proteins were visualized using an ECL detection system. *β*-Actin was used as an internal control. (c) and (d) The cell lysates from the cells treated with As_4_O_6_ were assayed for *in vitro* caspase-3, caspase-8, and caspase-9 activity using DEVD-pNA, IETD-pNA, and LEHD-pNA, respectively, as substrates. The released fluorescent products were measured. Each bar graph represents mean ± SD of three independent experiments. *P < 0.05 between the treated and the untreated control groups.

We then assessed the effects of As_4_O_6_ on caspases and their substrates (PARP and PLC*γ*-1). As_4_O_6_ decreased the expression levels of procaspase-3, procaspase-8, and procaspase-9 in a dose- and time-dependent manner. With the decrease of procaspases, the cleavages of PARP and PLC*γ*-1, the substrates of caspases, were found to be progressed in a dose- and time-dependent manner (Figures 2(a) and 2(b)). These findings suggest that As_4_O_6_ may induce apoptosis through caspase activation. To confirm and quantify the proteolytic activation of caspases, we assessed their activities using colorimetric assay kits. The caspase activity assay also showed that As_4_O_6_ increased proteolytic activities of caspases in a dose- and time-dependent manner (Figures 2(c) and 2(d)).

### 3.4. Effects of As_4_O_6_ on Bcl-2 Family Members and X-Linked Inhibitor of Apoptosis (XIAP)

**Figure 3:**
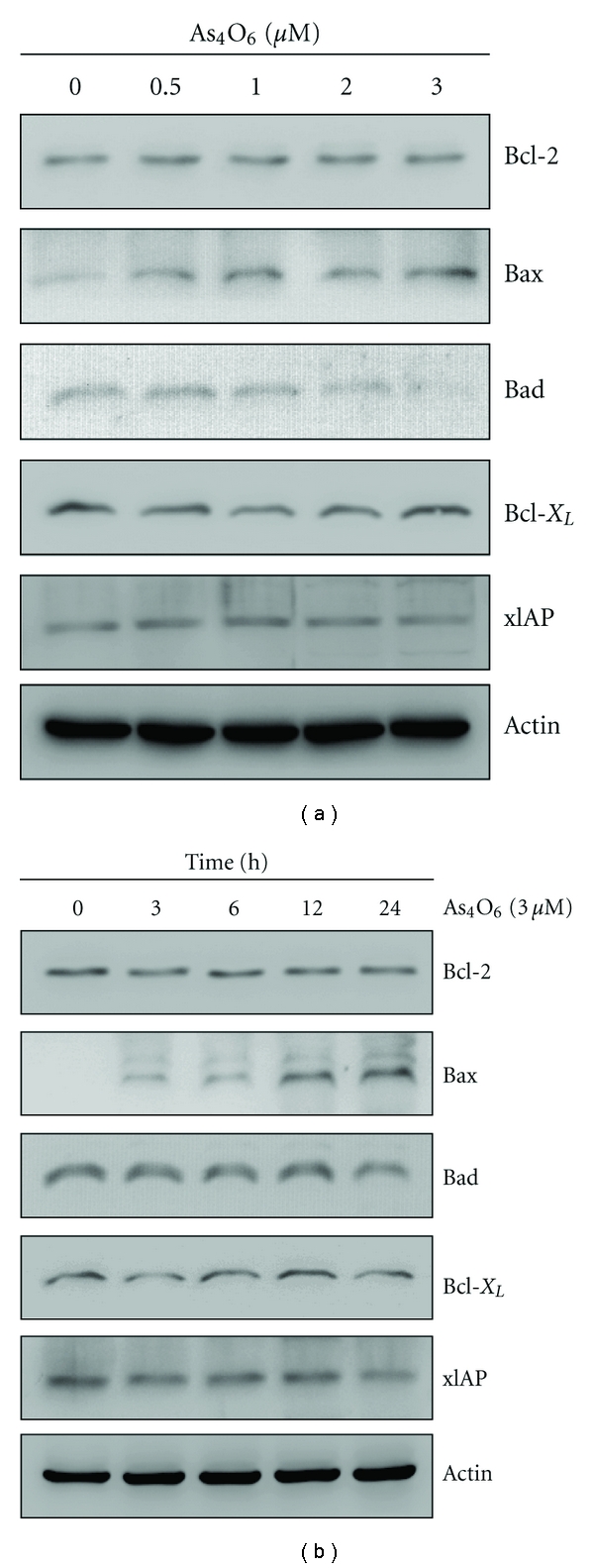
Regulation of Bcl-2 and IAP family proteins during As_4_O_6_-induced apoptosis. As_4_O_6_ increases the expressions of Bax in a dose- and time-dependent manner whereas the expressions of Bcl-2, Bad, Bcl-xL, and XIAP remain unchanged or slightly reduced. (a) The cells were treated with the indicated concentrations of As_4_O_6_ for 24 h. (b) The cells were treated with 3 **μ**M of As_4_O_6_ for the indicated times. The cells treated with As_4_O_6_ were lysed and equal amounts of proteins were then separated by SDS-polyacrylamide gels and transferred to nitrocellulose membranes. The membranes were probed with the indicated antibodies and detected by an ECL detection system. To confirm equal loading, the blot was stripped of the bound antibody and reprobed with the anti *β*-Actin antibody. The results are from one representative experiment of at least two independent experiments that showed similar patterns.

To elucidate further underlying mechanisms of As_4_O_6_-induced apoptosis, we assessed the levels of Bax, Bcl-2, Bad, Bcl-xL, and XIAP, which play a crucial role in apoptosis. Western blotting revealed that As_4_O_6_ induced an increase in the expressions of Bax (proapoptotic protein) in a dose- and time-dependent manner whereas the expression of Bcl-2, Bad, Bcl-xL, and XIAP (antiapoptotic proteins) remained unchanged or slightly reduced (Figure 3(a)). The induction of Bax expression began to clearly be observed at 12 hours after the treatment (Figure 3(b)). This finding suggested the possibility that the mechanism of Bax induction was related to the transcriptional activity. These findings suggested that upregulation of Bax protein and increased Bax/Bcl-2 ratio should be an important mechanism of As_4_O_6_-induced apoptosis in U937 cells.

### 3.5. Effects of As_4_O_6_ on Autophagy

**Figure 4:**
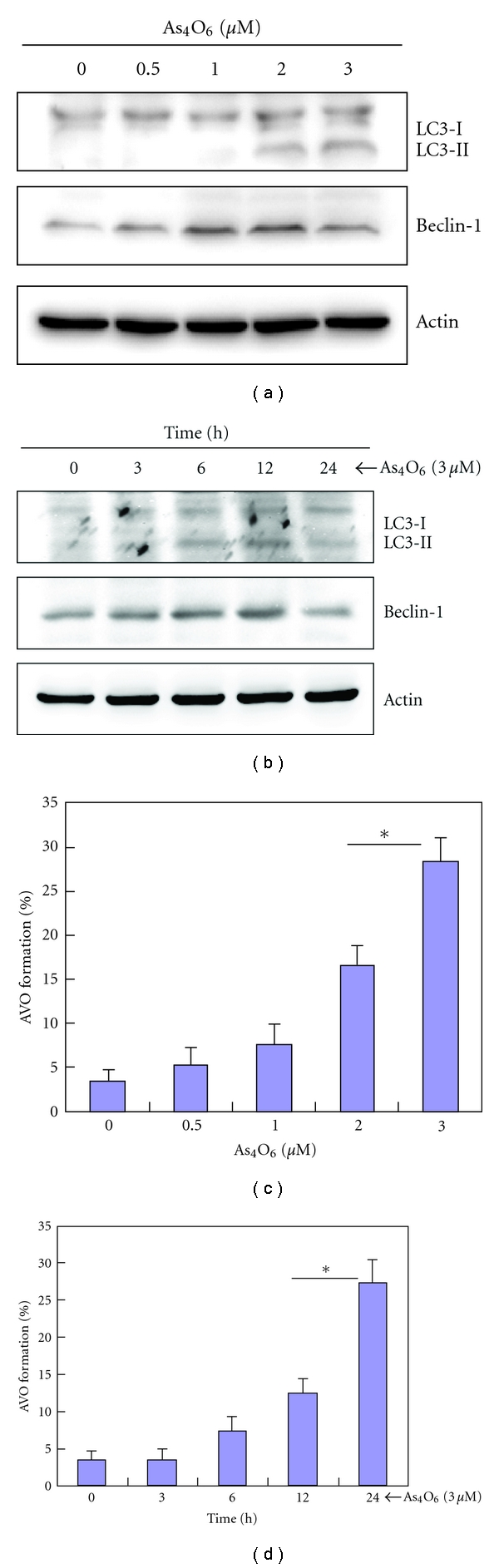
Effects of As_4_O_6_ on the autophagy in U937 cells. AVO formation by As_4_O_6_ is dose- and time-dependent. (a) and (c) The cells were treated with the indicated concentrations of As_4_O_6_ for 24 h. (b) and (d) The cells were treated with 3 **μ**M of As_4_O_6_ for the indicated times. (a) and (c) The cells treated with As_4_O_6_ were lysed and equal amounts of proteins were then separated by SDS-polyacrylamide gels and transferred to nitrocellulose membranes. The membranes were probed with the indicated antibodies and detected by an ECL detection system. To confirm equal loading, the blot was stripped of the bound antibody and reprobed with the anti *β*-Actin antibody. (b) and (d) The cells treated with As_4_O_6_ were stained with 5 **μ**g/mL acridine orange for 17 min and collected in phenol red-free growth medium. Green (510–530 nm) and red (650 nm) fluorescence emission illuminated with blue (488 nm) excitation light was measured with a FACSCalibur (Becton Dickinson).

Many studies have demonstrated that As_2_O_3_ can induce cell death through autophagy [13]. During autophagy, LC3-1 is converted to membrane-bound LC3-II that correlates with the extent of autophagosome formation which characterizes autophagy. For the autophagosome formation, Beclin-1 is important in mammalian cells. Hence, we assessed the expression of LC-3 (a marker for autophagy) and beclin-1 to check whether As_4_O_6_-induced cell death is involved in type II programmed cell death, autophgy. Western blotting revealed that As_4_O_6_ induced LC3 conversion (increase in the ratio of LC3-II/LC3-I) and increased the expressions of beclin-1 in a dose- and time-dependent manner (Figures 4(a) and 4(b)). The level of autophagosome formation corresponds with the ratio of LC3-II/LC3-I. Moreover, we also obtained evidence for As_4_O_6_-induced autophagy by measuring AVO formation through acridine orange staining. As shown in Figures 4(c) and 4(d), As_4_O_6_ induced the accumulation of AVO in a dose- and time-dependent manner.

### 3.6. Effects of Bcl-2 on As_4_O_6_-Induced Autophagy and Apoptosis

**Figure 5:**
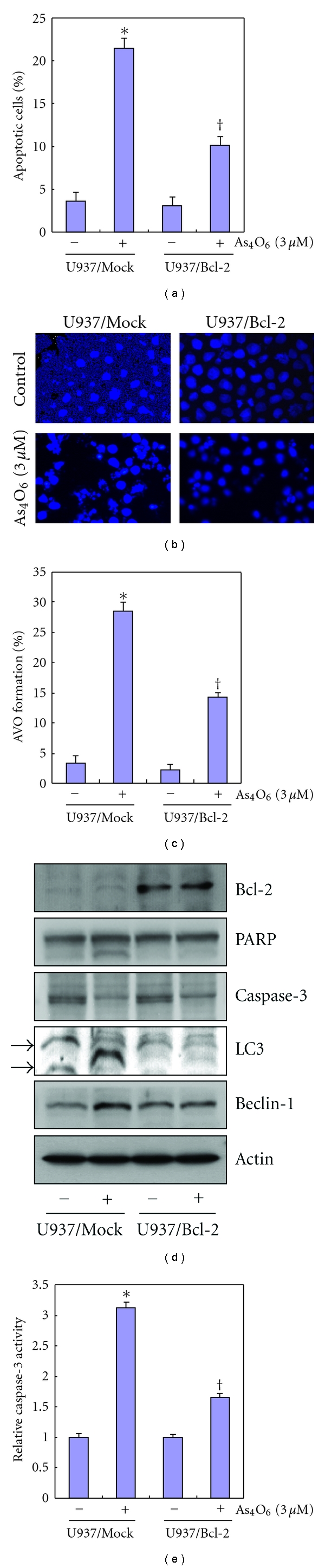
Effects of Bcl-2 overexpression in U937 cells on the apoptosis and autophagy induced by As_4_O_6_. Overexpression of Bcl-2 suppresses the induction of Beclin-1 and LC3 conversion in response to As_4_O_6_ as well as As_4_O_6_-induced caspase-3 activation and PARP cleavages (a) U937/vector or U937/Bcl-2 cells were treated with 3 **μ**M of As_4_O_6_ for 24 h. Sub-G1 DNA content was analyzed by flow cytometry. (b) To confirm apoptosis, the cells were stained with DAPI solution after fixation. Stained nuclei were then observed under fluorescent microscope using a blue filter (Magnification, X 400). (c) The cells treated with As_4_O_6_ were stained with 5 **μ**g/mL acridine orange for 17 min, and collected in phenol red-free growth medium. Green (510–530 nm) and red (650 nm) fluorescence emission illuminated with blue (488 nm) excitation light was measured with a FACSCalibur (Becton Dickinson). (d) The cells were lysed and equal amounts of proteins were then separated by SDS-polyacrylamide gels and transferred to nitrocellulose membranes. The membranes were probed with the indicated antibodies and detected by an ECL detection system. (e) The cell lysates from the cells treated with As_4_O_6_ were assayed for *in vitro* caspase-3activity using DEVD-pNA. The released fluorescent products were measured. The data are shown as means ± SD of three independent experiments. *P < 0.05 between the groups treated with and without As_4_O_6_, ^†^ *P* < 0.05 between the U937/vector and U937/Bcl-2 cells.

From the above, we found that As_4_O_6_ induced not only apoptosis through Bax induction but also autophgy through Beclin-1 induction. It has been suggested that the autophagy can be induced by apoptotic insults through up-regulation of Beclin-1. Bcl-2 is a well-known antiapoptotic molecule, and the interaction between Bcl-2 and Beclin-1 is important in the induction of autophgy. Therefore, we assessed Beclin-1 response to Bcl-2 overexpression and the effects of Bcl-2 overexpression on As_4_O_6_-induced autophgy and apoptosis by comparing those between U937/vector and U937/Bcl-2 cells that constitutively express high levels of Bcl-2. As shown in Figure 5(a), Bcl-2 overexpression led to significantly suppress the apoptosis induced by As_4_O_6_. We assessed the changes in nuclear morphology of As_4_O_6_-treated cells by DAPI staining. The DAPI staining showed that Bcl-2 overexpression reduced the frequency of condensed and fragmented nuclei in the As_4_O_6_-treated U937 cells which indicate apoptosis (Figure 5(b)). We also assessed the effects of Bcl-2 overexpression on As_4_O_6_-induced autophagosome formation. It reduced the As_4_O_6_-As_4_O_6_-induced AVO formation (Figure 5(c)). To confirm this finding at the molecular level, we performed western blotting for the molecules involved in As_4_O_6_-induced apoptosis and autophagy. It was observed on Western blotting that the overexpression of Bcl-2 suppressed the induction of Beclin-1 and LC3 conversion in response to As_4_O_6_, with the suppression of As_4_O_6_-induced caspase-3 activation and PARP cleavages (Figures 5(d) and 5(e)). These findings suggested that the increased Bcl-2 should significantly influence the antitumor effects of As_4_O_6 _through suppressing autophagy as well as apoptosis, and that Beclin-1 induction by As_4_O_6_ might be related to apoptosis induction.

### 3.7. Inhibition of As_4_O_6_-Induced Apoptosis and Autophagy in U937 Cells by N-Acetylcysteine (NAC)

**Figure 6:**
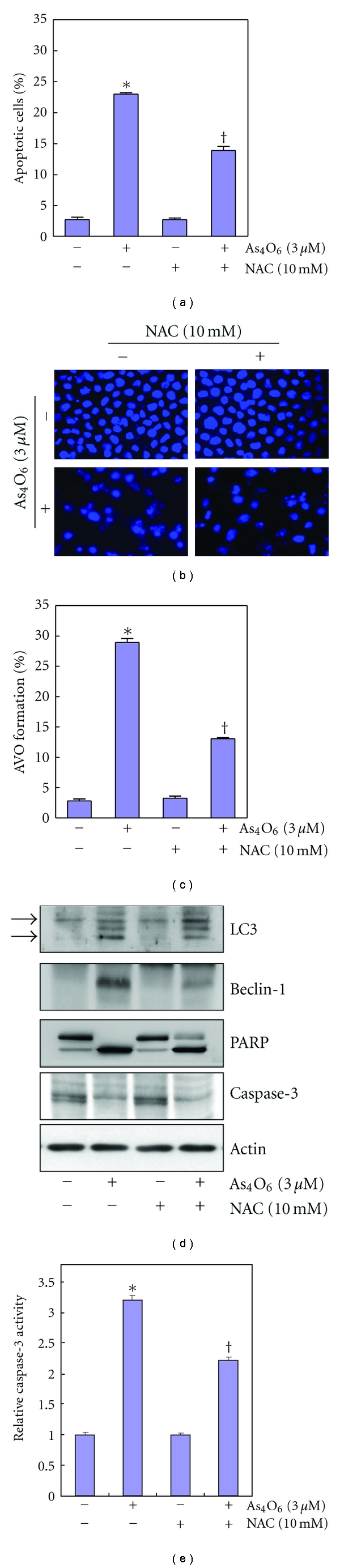
Inhibition of As_4_O_6_-induced apoptosis and autophagy in U937 cells by N-acetylcysteine (NAC). NAC reduces the As_4_O_6_-induced autophagosome formation as well as As_4_O_6_-induced cell death. (a) U937 cells were treated with NAC (10 mM) 30 min before As_4_O_6_ (3 **μ**M) for 24 h. The cells treated with As_4_O_6_ were stained with 5 **μ**g/mL acridine orange for 17 min and collected in phenol red-free growth medium. Green (510–530 nm) and red (650 nm) fluorescence emission illuminated with blue (488 nm) excitation light was measured with a FACSCalibur (Becton Dickinson). (b) Sub-G1 DNA content was analyzed by flow cytometry. (c) To confirm apoptosis, the cells were stained with DAPI solution after fixation. Stained nuclei were then observed under fluorescent microscope using a blue filter (Magnification, X 400). (d) The cells were lysed and equal amount of the lysate was separated by SDS-polyacrylamide gels and then transferred to nitrocellulose membranes. The membranes were probed with the indicated antibodies and detected by an ECL detection system. To confirm equal loading, the blot was stripped of the bound antibody and reprobed with the anti *β*-Actin antibody. (e) The cell lysates from the cells treated with As_4_O_6_ were assayed for *in vitro* caspase-3activity using DEVD-pNA. The released fluorescent products were measured. The data are shown as means ± SD of three independent experiments. *P < 0.05 between the groups treated with and without As_4_O_6_, ^†^ *P* < 0.05 between the groups treated with and without NAC.

A previous study showed that As_4_O_6_ induced reactive oxygen species (ROS) leading to loss of mitochondrial potential (MMP, *ΔΨm*) [14]. In addition, As_2_O_3_ induced apoptosis in leukemic cell lines via modulation of the glutathione (GSH) redox system [15]. NAC is an antioxidant that functions by donating a cysteine to the de novo synthesis of GSH. To assess the effects of NAC on As_4_O_6_-induced autophgy and apoptosis, we analyzed the cells with sub-G1 DNA content and AVOs using flow cytometry after As_4_O_6_ treatment and observed changes in nuclear morphology of As_4_O_6_-treated cells by DAPI staining. We found that NAC reduced the As_4_O_6_-induced autophagosome formation as well as As_4_O_6_-induced cell death (Figures 6(a) and 6(b)). The DAPI staining revealed that NAC reduced the frequency of condensed and fragmented nuclei in the As_4_O_6_-treated U937 cells (Figure 6(c)).

To confirm this finding at the molecular level and determine whether the Beclin-1-induction is associated with ROS production, we performed western blotting for the molecules involved in As_4_O_6_-induced apoptosis and autophagy. Western blotting revealed that NAC suppressed As_4_O_6_-induced Beclin-1 induction and LC3 conversion and As_4_O_6_-induced caspase-3 activation and PARP cleavages (Figures 6(d) and 6(e)). These findings suggested that the As_4_O_6_-induced autophagy as well as apopotosis should be related to ROS production. These findings suggested that ROS production by As_4_O_6_ should be related to Beclin-1-induced autophagy as well as apoptosis.

## 4. Discussion

This study was designed to determine whether As_4_O_6_ has anticancer properties in human leukemic cells and further to investigate the underlying mechanisms as compared to that of the anticancer effects of As_2_O_3_. Regarding the As_4_O_6_-induced cell death, it has not been reported that autophagic cell death is a critical mechanism for the effects. To gain insights into the mechanisms for As_4_O_6_-induced cell death, we investigated the both apoptosis and autophagy. Here, we found that As_4_O_6_ did not only induce caspase-dependent apoptotic cell death but also induce autophagic cell death. Arsenic trioxide (As_2_O_3_) is well known to have anticancer properties against leukemic cells as well as other cancer cells. The reported mechanisms of As_2_O_3_-induced cell death vary depending on the cell lines: caspase-dependent apoptosis [16, 17], caspase-independent [18], and autophagic cell death [13, 19]. Even in the studies on As_2_O_3_-induced cell death of U937 cells, some studies reported that caspase-dependent apoptosis is a major mechanism for the cell death [20] and other studies suggested that autophagic cell death is a critical mechanism for the antileukemic effects [13]. In other leukemic cell lines, arsenic trioxide did not only induce apoptosis but also induced autophagic cell death in leukemia cell lines via upregulation of Beclin-1 [21]. The mechanism for As_2_O_3_-induced cell death appears similar to that of As_4_O_6_ although there is a report showing a significant difference between As_2_O_3_- and As_4_O_6_-induced cell death [10]. 

Apoptosis is the process of programmed cell death that can be executed through extrinsic pathway and intrinsic pathway. Either pathway is involved in mitochondrial outer membrane permeabilization which is a critical event in apoptosis [22]. The mitochondrial outer membrane permeabilization is controlled by several factors, such as the Bcl-2 and IAP protein family. The Bcl-2 family consists of proapoptotic factors (e.g., Bax, Bad, etc.) and antiapoptotic factors (e.g., Bcl-2, Bcl-xL, etc.). The Bax/Bcl-2 ratio is known as a key factor in triggering the apoptotic process. We found that caspase-dependent apoptosis was one of mechanisms for the antileukemic effects of As_4_O_6_ through the induction of Bax protein. At first we were puzzled at this result (Bax induction by As_4_O_6_) in p53-deficient U937 cells because tumor suppressor p53 plays the central role in regulating Bax protein, a proapoptotic protein. However, the previous report that Bax protein can be induced in U937 cells through the transaction of p73 gene can explain our results [23]. 

This study also suggested that the Beclin-1-induced autophagic cell death could be another mechanism for As_4_O_6_-induced cell death. This finding showing As_4_O_6_-induced autophagy in As_4_O_6_-induced cell death is also similar to that in As_2_O_3_-induced leukemic cell death [13, 21]. Recently it has been reported that arsenic trioxide induces a Beclin-1-independent autophagic cell death in ovarian cancer cells [24]. This finding suggested that mechanisms of As_2_O_3_-induced cell death should vary depending on the cell lines; so it is not unknown whether our results are applicable to other cancer cells. Therefore, we are going to investigate the mechanism for As_4_O_6_-induced cell death in other solid cancer cells. Our results were derived from a single leukemic cell line; so it is difficult to generalize this finding to all leukemic cells. However, those indicated that As_4_O_6_-induced Beclin-1 induction which led to autophagy can be another mechanism for its antileukemic effects on U937 cells. 

Another limitation is that we have not verified yet whether Beclin-1-induced autophagy is a critical mechanism for As_4_O_6_-induced cell death or a mechanism to rescue cancer cells from toxic damage. Now that the autophagic cell death is mainly a morphologic definition (i.e., cell death associated with autophagosomes/autolysosomes), there is still no definite evidence that a specific mechanism for autophagic death actually exists. Nonetheless, it is quite conceivable that the autophagy induced by As_4_O_6_ could eventually destroy a cell because it has been reported that autophagic cell death is a major mechanism for the anticancer activities of radiation [25] and temozolomide [26] as well as arsenic compounds [13, 21]. 

Unlike As_2_O_3_-induced cell death in U937 cells, As_4_O_6_ did not suppress Bcl-2 expression in this study, but we tested the effects of augmented Bcl-2 on apoptosis and autophagy as well as apoptosis induced by As_4_O_6_. We observed that augmented Bcl-2 significantly suppressed the autophagic cell death as well as apoptotic cell death induced by As_4_O_6_. This finding is consistent with the previous study [27–29]. 

In aerobic organisms ROS is produced in the mitochondria via the electron transport chain during energy production. Under normal circumstances, reductive enzymes such as catalase and superoxide dismutase can defend cells from the ROS damage, but if ROS is produced high enough to cause severe cellular damage, a cell may undergo programmed cell death [20, 30]. We observed that NAC suppressed As_4_O_6_-induced autophagy as well as As_4_O_6_-induced apoptosis. This finding suggested that ROS production should be greatly involved in As_4_O_6_-induced autophagy as well as As_4_O_6_-induced apoptosis. Although the possibility that Beclin-1-induced autophagy can be a process to rescue cancer cells from As_4_O_6_-induced apoptosis could not be excluded, our finding suggested that ROS induced by As_4_O_6_ should lead to Beclin-1-induced autophagy. 

In conclusion, we have demonstrated that As_4_O_6_-induced cell death is carried on through Beclin-1-induced autophagic cell death as well as caspase-dependent apoptosis, and that the ROS production by As_4_O_6_ plays important roles in triggering both Beclin-1-induced autophagic cell death and caspase-dependent apoptosis. This study provides evidence that As_4_O_6_-induced cell death is related to Beclin-1-induced autophagy as well as caspase-dependent apoptosis and As_4_O_6_ might be an effective agent for the treatment of leukemia similar to As_2_O_3_.
